# Deficiency of the metabolic enzyme SCHAD in pancreatic β-cells promotes amino acid–sensitive hypoglycemia

**DOI:** 10.1016/j.jbc.2023.104986

**Published:** 2023-06-29

**Authors:** Johanna L. St-Louis, Khadija El Jellas, Kelly Velasco, Brittany A. Slipp, Jiang Hu, Geir Helgeland, Solrun J. Steine, Dario F. De Jesus, Rohit N. Kulkarni, Anders Molven

**Affiliations:** 1 Section on Islet Cell and Regenerative Biology, Joslin Diabetes Center, Harvard Medical School, Boston, USA; 2 Department of Clinical Medicine, Gade Laboratory for Pathology, University of Bergen, Bergen, Norway; 3 Department of Medicine, Beth Israel Deaconess Medical Center, Harvard Medical School, Boston, USA; 4 Harvard Stem Cell Institute, Boston, USA; 5 Department of Pathology, Haukeland University Hospital, Bergen, Norway; 6 Section for Cancer Genomics, Haukeland University Hospital, Bergen, Norway

**Keywords:** congenital hyperinsulinism of infancy, hypoglycemia, β-cell, short-chain 3-hydroxyacyl-CoA dehydrogenase, SCHAD, *HADH*, amino acids, insulin secretion, knockout mouse model, β-cell dedifferentiation, transcriptomics, islets

## Abstract

Congenital hyperinsulinism of infancy (CHI) can be caused by a deficiency of the ubiquitously expressed enzyme short-chain 3-hydroxyacyl-CoA dehydrogenase (SCHAD). To test the hypothesis that SCHAD-CHI arises from a specific defect in pancreatic β-cells, we created genetically engineered β-cell-specific (β-SKO) or hepatocyte-specific (L-SKO) SCHAD knockout mice. While L-SKO mice were normoglycemic, plasma glucose in β-SKO animals was significantly reduced in the random-fed state, after overnight fasting, and following refeeding. The hypoglycemic phenotype was exacerbated when the mice were fed a diet enriched in leucine, glutamine, and alanine. Intraperitoneal injection of these three amino acids led to a rapid elevation in insulin levels in β-SKO mice compared to controls. Consistently, treating isolated β-SKO islets with the amino acid mixture potently enhanced insulin secretion compared to controls in a low-glucose environment. RNA sequencing of β-SKO islets revealed reduced transcription of β-cell identity genes and upregulation of genes involved in oxidative phosphorylation, protein metabolism, and Ca^2+^ handling. The β-SKO mouse offers a useful model to interrogate the intra-islet heterogeneity of amino acid sensing given the very variable expression levels of SCHAD within different hormonal cells, with high levels in β- and δ-cells and virtually absent α-cell expression. We conclude that the lack of SCHAD protein in β-cells results in a hypoglycemic phenotype characterized by increased sensitivity to amino acid-stimulated insulin secretion and loss of β-cell identity.

Congenital hyperinsulinism of infancy (CHI) is a rare, inherited condition that is characterized by persistent hypoglycemia and inappropriately elevated insulin secretion (1). It is caused by mutations in genes that affect pancreatic β-cell function by directly or indirectly impacting the insulin secretion pathway (2). Most cases of CHI are “channelopathies” caused by mutations in the β-cell K_ATP_ channel genes *ABCC8* and *KCNJ11*. Some CHI cases can be classified as “metabolopathies” because there is an underlying defect in metabolic enzymes such as glucokinase and glutamate dehydrogenase (GDH) (2). Another metabolic enzyme associated with CHI is short-chain 3-hydroxyacyl-CoA dehydrogenase (SCHAD), encoded by the *HADH* gene (3, 4). SCHAD is an enzyme of the mitochondrial matrix that catalyzes the third of four steps in the cyclic series of fatty acid-degrading enzymatic reactions (5).

Besides its role in fatty acid oxidation, SCHAD has a second function that may be unique to the β-cell. This became clear following the discovery that recessive loss-of-function mutations in the *HADH* gene cause protein-sensitive, diazoxide-responsive CHI with elevated levels of plasma 3-hydroxybutyryl-carnitine and urine 3-hydroxyglutaric acid (3, 4, 6). Surprisingly, fatty acid metabolism appeared unaffected and symptoms typical for fatty acid oxidation defects were generally not observed in the patients. Thus, there was a discrepancy between SCHAD’s role in fatty acid oxidation and the hyperinsulinemic phenotype caused by a disrupted *HADH* gene. However, SCHAD had been observed to bind GDH in mitochondrial extracts (7) and by studying a mouse model of general SCHAD deficiency (SCHADKO), Li *et al.* (8) suggested that the SCHAD protein is an inhibitory regulator of GDH. Mutations in the gene encoding GDH can also cause CHI, but in contrast to SCHAD-CHI, they result in an overactive enzyme and act dominantly (9, 10).

SCHAD and GDH are both ubiquitously expressed enzymes. However, SCHAD expression is particularly high in mature β-cells compared to other cell types and also to other fatty acid oxidation enzymes, indicating a β-cell specific function (11, 12). Intriguingly, the gene is one of top ten markers defining human β-cell identity in a recent meta-analysis of transcriptomic data (13). We previously investigated whether SCHAD deficiency in the endocrine pancreas is sufficient to cause hypoglycemia. We found that islets of Langerhans from SCHADKO mice, when transplanted under the kidney capsule of streptozotocin-induced diabetic mice, were associated with significantly lower blood glucose levels compared to islets transplanted from control animals (14). While that study highlighted the importance of SCHAD expression within the islets, it did not distinguish between different islet cell populations. Thus, whether SCHAD-CHI has a specific β-cell etiology or if intra-islet crosstalk also contributes is still unknown. Furthermore, the transplantation approach does not allow for detailed screening of organ systems and cannot serve as an appropriate model for studying SCHAD deficiency in normal physiology.

To circumvent these limitations and to directly address the tissue-specific roles of SCHAD, we generated a conditional knockout model of SCHAD by introducing LoxP sites in the murine *Hadh* gene. These “floxed” mice were crossed with mice expressing either Ins1-Cre or Alb-Cre to eliminate SCHAD expression specifically in β-cells or hepatocytes, respectively. The metabolic phenotype of the cell-specific knock-out models was then analyzed to elucidate the role of SCHAD in glucose metabolism.

## Results

### Generation of β-cell-specific SCHAD knockout mice

*Hadh*^lox/lox^ mice were generated as described under Experimental procedures. To delete SCHAD expression specifically from β-cells, *Hadh*^lox/lox^ mice were crossed with Ins1-Cre mice (15). To ensure that neither the introduction of LoxP sites around exon 3 of the *Hadh* gene nor the disruption of one allele of the *Ins1* gene in Ins1-Cre mice affected glucose homeostasis, we compared bodyweights, glucose levels, and glucose tolerance of *Hadh*^lox/lox^ and Ins1-Cre mice to wildtype C57BL/6 mice. No significant phenotypic differences were detected between genotypes (data not shown). After introducing Ins1-Cre in the floxed *Hadh*^lox/lox^ strain, we, therefore, established two experimental groups: *Hadh*^lox/lox^ animals that were either heterozygous for Ins1-Cre (β-SKO mice) or wildtype for this construct (littermate controls).

**Figure 1:**
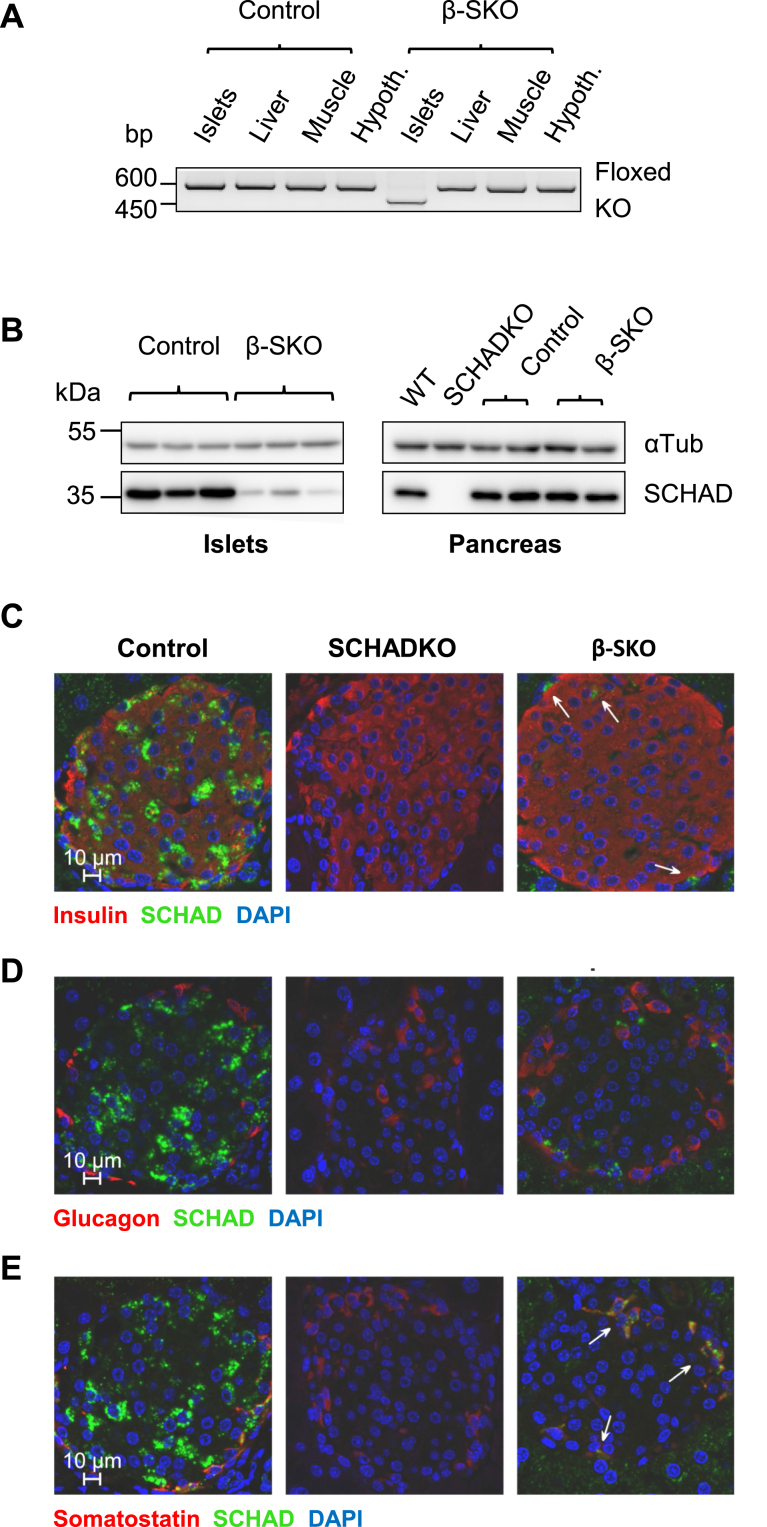
**Validation of disrupted SCHAD expression in β-SKO mice.***A*, PCR assay and agarose gel electrophoresis for detection of the floxed and knockout *Hadh* alleles in islets, liver, skeletal muscle, and hypothalamus of littermate control and β-SKO mice. Recombination was assessed using the primers P1, P2, and P3 described in the Experimental procedures section. The P1-P2 combination yields a PCR product of 600 bp length only when exon 3, which includes the binding site for P2, is intact. The P1-P3 combination yields a 450 bp PCR product only when exon 3 is deleted. *B*, Western blots for detection of SCHAD protein expression in lysates of isolated islets and whole pancreas of control and β-SKO mice. Lysates from wild-type mice (WT) and the global *Hadh* knockout mouse (SCHADKO) were included as positive and negative controls, respectively. Detection of tubulin alpha-1A chain (αTub) served as loading control. *C*, immunohistochemistry performed on pancreas sections using antibodies against insulin (*red*) or SCHAD (*green*). Representative islets from control, SCHADKO, and β-SKO mice are shown. *White arrows* indicate SCHAD-positive cells in a β-SKO islet. Nuclei are stained blue with DAPI. *D*, same as (*C*) using antibodies against glucagon (*red*) and SCHAD (*green*). *E*, same as (*C*) using antibodies against somatostatin (*red*) and SCHAD (*green*). White arrows indicate cells that are positive both for SCHAD and somatostatin in a β-SKO islet.

The successful disruption of the *Hadh* gene by Cre-mediated recombination and reduction of SCHAD protein expression in β-SKO islets was confirmed by PCR and Western blotting (Fig. 1, *A* and *B*). Indeed, only DNA derived from islet preparations yielded the recombination-specific 450 bp band, while liver, skeletal muscle, and hypothalamus showed only the product for intact *Hadh*^*lox/lox*^ (Fig. 1*A*). Western blotting confirmed a marked reduction of SCHAD protein levels in samples from isolated islets but not in the whole pancreas (Fig. 1*B*). Skeletal muscle, liver, or hypothalamus did not show alterations in SCHAD protein levels (Fig. S1*B*). Thus, *Hadh* knockout was detected only in samples containing β-cells. The residual floxed PCR product and SCHAD protein expression in islets of β-SKO mice can be explained by the presence of other islet cell types and contaminating exocrine cells in islet preparations.

### SCHAD is highly expressed in pancreatic δ-cells

To further demonstrate the absence of SCHAD expression specifically in β-cells of β-SKO mice, we co-immunostained pancreas sections from control, SCHADKO (global knockout), and β-SKO mice for SCHAD protein and the islet cell hormones insulin (β-cells), glucagon (α-cells) or somatostatin (SST) (δ-cells) (Fig. 1, *C*–*E*). Pancreas sections from control mice exhibited SCHAD staining with higher intensity in the endocrine compared to the exocrine pancreas. No signal was detected in sections from SCHADKO animals. β-SKO sections, on the other hand, showed a widespread absence of SCHAD expression only in islets, with a few positive cells in areas surrounding the islet core. Co-immunostaining for glucagon or SST showed that the remaining SCHAD-expressing cells stained strongly for SST and were negative for glucagon (Fig. 1, *D* and *E*). These results indicated a specific knockout of SCHAD in β-cells and suggested that endogenous expression of SCHAD is high in δ-cells and virtually absent in α-cells.

### β-cell-specific SCHAD deficiency leads to reduced blood glucose levels

**Figure 2:**
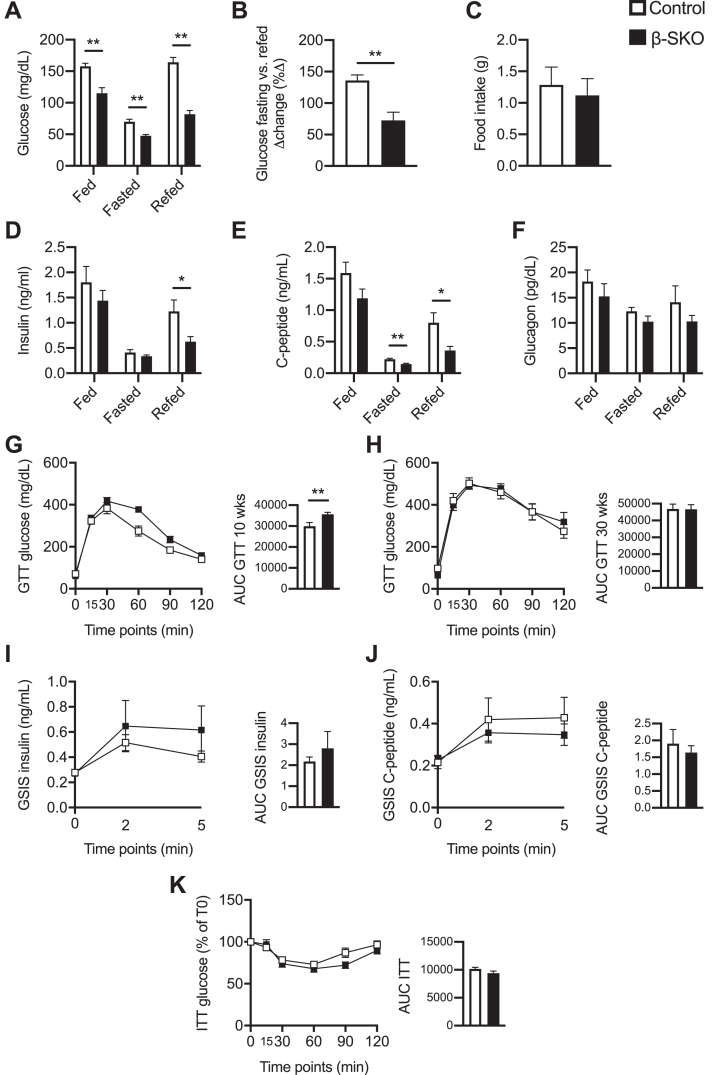
**Glucose homeostasis and insulin tolerance in male control and β-SKO mice.***A*, plasma glucose in fed, 16-h fasted, and refed 13-week-old control and β-SKO mice (n = 6–10). *B*, difference (Δ) in percentage change of fasted *versus* refed plasma glucose in control and β-SKO mice (n = 6–7). *C*, food intake per mouse during the 4-h refeeding period in control and β-SKO mice (n = 5–7). *D*–*F*, plasma insulin (n = 7–10), C-peptide (n = 8–10), and glucagon (n = 7) in fed, 16-h fasted and refed control, and β-SKO mice (13 weeks old). *G*, GTT and AUC (area under the curve) of 10-week-old control and β-SKO mice (n = 10–11). *H*, same as (*G*) for 30-week-old mice (n = 9). *I*, GSIS and AUC of 12-week-old control and β-SKO mice (n = 10). *J*, plasma C-peptide levels for the mice in (*I*) (n = 10). *K*, ITT and AUC of 10-week-old control and β-SKO mice (n = 9). All data are represented as mean ± SEM. *p*-values: ∗ < 0.05, ∗∗ < 0.005.

To investigate whether β-cell-specific knockout of SCHAD is sufficient to cause hypoglycemia, we compared body weight and glucose homeostasis between controls and β-SKO mice. Body and tissue weights were not significantly affected by β-cell SCHAD deficiency, neither in male nor in female mice (Fig. S2). Assessment of glucose homeostasis showed that male β-SKO mice generally displayed a reduction in plasma glucose levels compared to control mice in the random-fed states, after 16 h fasting, or after 4 h of subsequent re-feeding (control *versus* β-SKO; random-fed: 157.9 ± 4.6 *versus* 115.2 ± 8.8 mg/dl, *p* = 0.0016; 16 h fasted: 70 ± 3.8 *versus* 47.7 ± 2.9 mg/dl, *p* = 0.0012; 4 h refed: 164.3 ± 18 *versus* 81.7 ± 6.1 mg/dl, *p* = 0.0012) (Fig. 2*A*). The increase in plasma glucose after re-feeding was strongly blunted in male β-SKO mice (control *versus* β-SKO; %Δ 136.1 ± 8.7 *versus* 72.6 ± 13.1 mg/dl, *p* = 0.005) (Fig. 2*B*), whereas food intake was not different from controls (Fig. 2*C*). Very similar results were obtained for females (Fig. S3, *A*–*C*).

**Figure 3:**
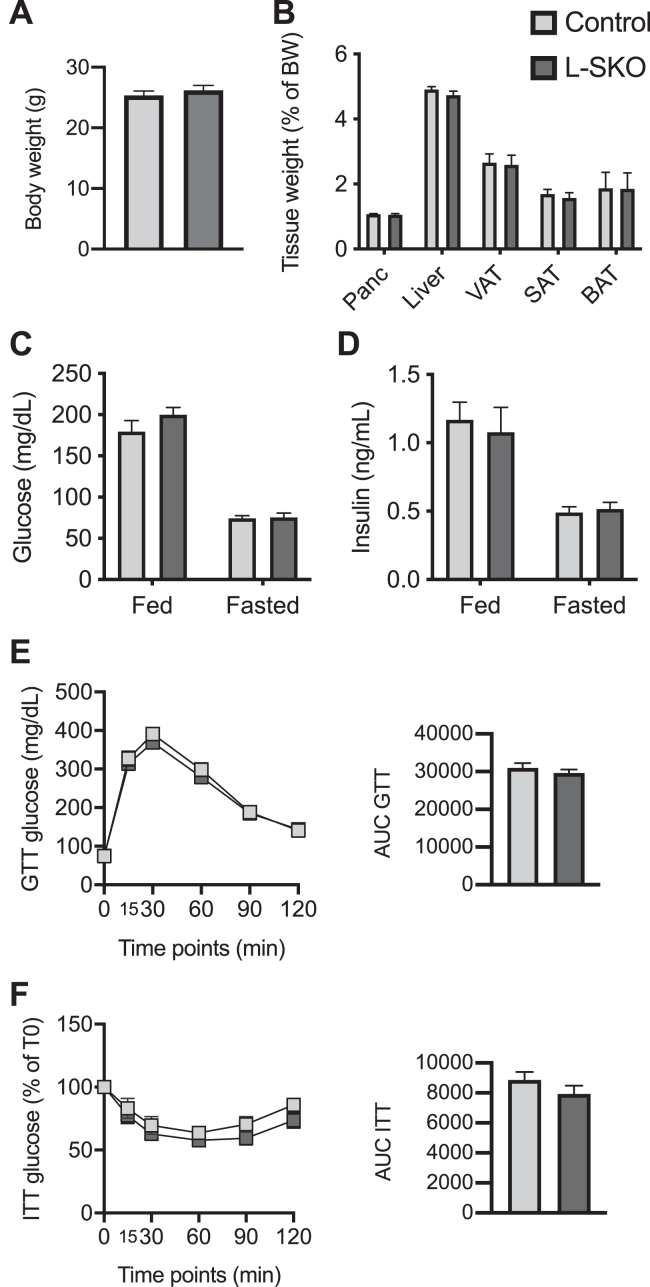
**Body and organ weights and glucose homeostasis of male control and L-SKO mice.***A*, bodyweight of 10-week-old control and L-SKO mice (n = 9–10). *B*, tissue weights of pancreas, liver, visceral (VAT), subcutaneous (SAT), and *brown* (BAT) adipose tissue as a percentage of body weight of 14-week-old control and L-SKO mice (n = 9–10). *C* and *D*, plasma glucose (n = 9–10) and insulin (n = 7–10) in fed and 16-h fasted control and L-SKO mice (12 weeks old). *E*, GTT and AUC of 10-week-old control and L-SKO mice (n = 9–10). *F*, ITT and AUC of 10-week-old control and L-SKO mice (n = 9–10). All data are represented as mean ± SEM.

The changes in circulating insulin generally followed the trends in glucose levels with a decrease in the fasting state and an increase in the refed state in both sexes. Surprisingly, plasma insulin levels were significantly lower in male β-SKO mice in the 4 h refed condition (control *versus*β-SKO; 1.2 ± 0.2 *versus* 0.6 ± 0.1 ng/ml, *p* = 0.04) (Fig. 2*D*). This was consistent with a reduction in C-peptide levels (16 h fasted; controls *versus*β-SKO; 0.2 ± 0.02 *versus* 0.1 ± 0.01 ng/ml, *p* = 0.003; 4 h refed: 0.8 ± 0.2 *versus* 0.4 ± 0.07 ng/ml, *p* = 0.03) (Fig. 2*E*). In contrast, no differences in plasma insulin or C-peptide levels were evident between the two female groups (Fig. 3, *D* and *E*). Glucagon levels did not show differences between genotypes in males and did not increase following a 16 h fast (Fig. 2*F*). In females, glucagon levels in both control and β-SKO mice increased following the 16-h fast and remained elevated after refeeding. However, there were no differences between genotypes (Fig. S3*F*).

Glucose tolerance in male, 10-week-old β-SKO mice was significantly impaired (AUC 29888 ± 1846 *versus* 35,584 ± 954, *p* = 0.004), but the difference disappeared when the mice reached 30 weeks of age (AUC 46867 ± 2846 *versus* 46,682 ± 2777, *p* = 0.7) (Fig. 2, *G* and *H*). Female mice showed no differences in GTT between groups (Fig. S3, *G* and *H*). GSIS and ITT were similar between groups in males and females (Fig. 2, *I*–*K*; Fig. S3, *I*–*K*). In summary, the plasma glucose levels of the β-SKO mouse were consistently decreased with little difference in hormone levels or tolerance tests when compared with control littermates.

### Glucose homeostasis is not affected in mice with hepatocyte SCHAD deficiency

The liver regulates plasma glucose levels *via* glycogenolysis and gluconeogenesis. A defect in these processes could result in hypoglycemia. SCHAD has been suggested to have liver-specific interactions with proteins involved in the urea cycle and ketone-body formation (16). Therefore, SCHAD deficiency could lead to defects in overall hepatocyte function and contribute to the hypoglycemic phenotype. To exclude the contribution of liver metabolism to the hypoglycemia seen in SCHAD deficiency, we studied L-SKO mice generated by crossing *Hadh*^lox/lox^ with Alb-Cre mice (17). Successful disruption of the *Hadh* gene by Cre-recombinase was confirmed by PCR and Western blot (Fig. S1, *C* and *D*). The 450 bp band corresponding to recombined *Hadh* was detected in liver DNA samples only from mice expressing Alb-Cre, and SCHAD protein was not detected in liver preparations from L-SKO mice by Western blot, indicating efficient deletion of SCHAD expression in hepatocytes.

Similar to the β-SKO animals, male and female L-SKO mice showed no significant differences in body or metabolic tissue weights when compared to littermate controls (Fig. 3, *A* and *B*; Fig. S4, *A* and *B*). Plasma glucose and insulin levels were elevated in the fed compared to the 16 h fasted state in all groups but not significantly different between control and L-SKO mice (Fig. 3, *C* and *D*; Fig. S4, *C* and *D*). Moreover, the mice displayed no differences in glucose tolerance or insulin sensitivity (Fig. 3, *E* and *F*; Fig. S4, *E* and *F*). Thus, in contrast to the β-cell-specific defect, we conclude that a lack of SCHAD in hepatocytes resulted in no overt phenotype related to glucose homeostasis.

### Amino acids worsen the glucose-lowering effects of β-cell SCHAD depletion and enhance insulin secretion in β-SKO mice and isolated β-SKO islets

Patients with SCHAD-CHI are known to be protein-sensitive and in islets from the global SCHADKO mouse, a combination of the amino acids leucine, glutamine, and alanine was sufficient to obtain maximal insulin secretion (8). We, therefore, fed a cohort of male control and β-SKO mice a diet with increased content of these amino acids (enriched diet, ED). We compared their metabolic phenotype to that of mice fed two control diets, eventually pooled into one common control group (ND, see Experimental procedures).

**Figure 4:**
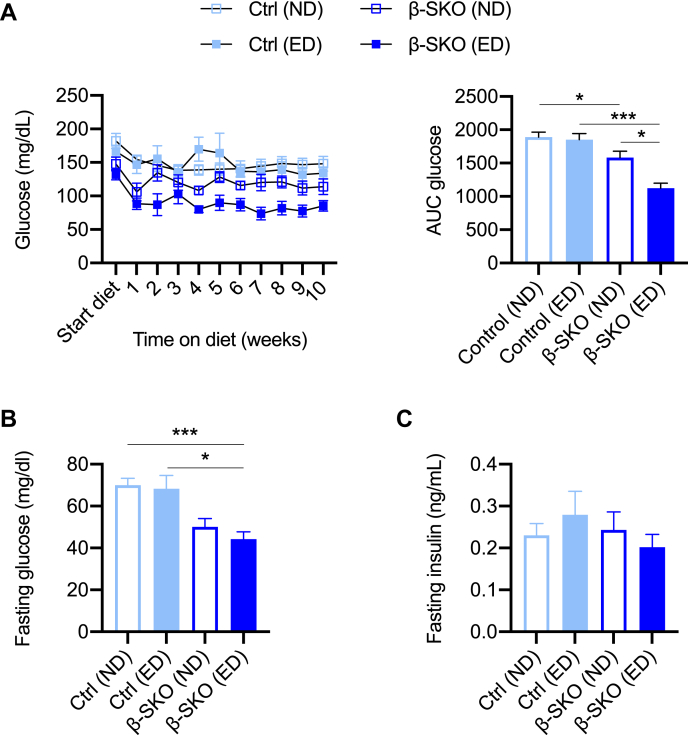
**Effect of amino acids on glucose homeostasis in the β-SKO model.***A*, random-fed plasma glucose levels and AUC of male control and β-SKO mice over the course of 10 weeks on a diet enriched in the amino acids alanine, glutamine and leucine (ED) compared with mice fed a normal diet (ND) (n = 6–9). *B*, fasting (16 h) plasma glucose levels of male control and β-SKO mice fed ED or ND for 14 weeks (n = 5–9). *C*, plasma insulin levels for the mice in (*B*). All data are represented as mean ± SEM. *p*-values: ∗ < 0.05, ∗∗∗ < 0.0005.

Placing the animals on the ED for 10 weeks did not significantly alter the body weights of control or β-SKO mice (Fig. S5*A*). Moreover, plasma glucose levels in control mice were not affected by ED compared to ND during this time period (Fig. 4*A*). Consistent with our previous experiments using normal chow (Fig. 2*A*), β-SKO mice fed ND exhibited lower plasma glucose levels compared to controls on the same diet (β-SKO *versus* control; AUC 1582 ± 272 *versus* 1893 ± 216 mg/dl, *p* = 0.044) (Fig. 4*A*). This reduction was exacerbated by administering ED to β-SKO mice: these animals exhibited significantly lower glucose levels both when compared to ED-fed controls (AUC 1125 ± 180 mg/dl *versus* 1852 ± 221 mg/dl, *p* = 0.0001) or when compared to ND-fed β-SKO mice (AUC 1125 ± 180 mg/dl *versus* 1582 ± 272, *p* = 0.035) (Fig. 4*A*). Fasted and randomly fed insulin levels were similar between groups despite lower plasma glucose in fasted and fed β-SKO mice on ND or ED compared to their respective control groups (Fig. 4, *B* and *C*; Fig. S5, *B* and *C*). Furthermore, glucose tolerance and GSIS were not significantly altered in ED-fed β-SKO mice when compared to the ND-fed β-SKO animals or ED-fed controls (Fig. S5, *D* and *E*).

**Figure 5:**
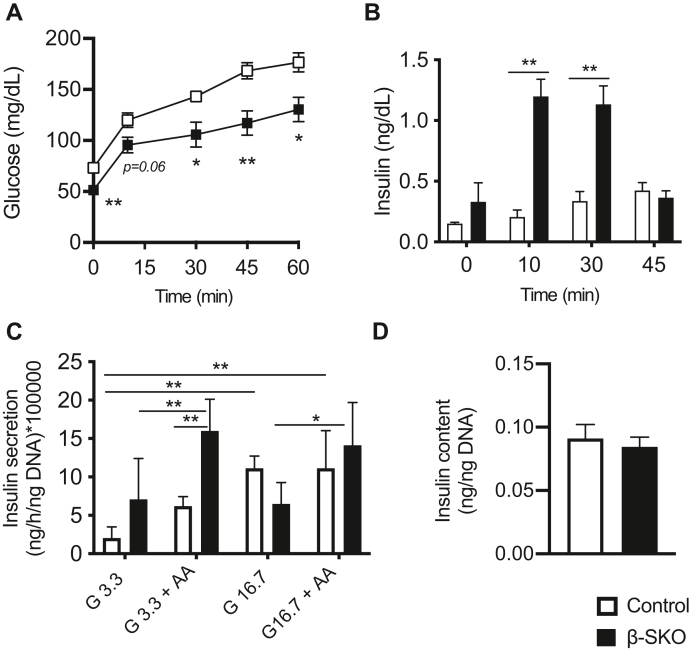
**Effect of amino acids on insulin secretion in β-SKO animals and isolated islets.***A*, plasma glucose from intraperitoneal amino acid administration on 16-h fasted β-SKO (n = 9) and control (n = 10) mice. *B*, plasma insulin levels before and after the amino acid injection. *C*, insulin secretion from isolated control and β-SKO islets stimulated with 3.3 mM glucose (G3.3), G3.3 in combination with amino acid mixture (AA), 16.7 mM glucose (G16.7), or G16.7 in combination with AA. Data are multiplied by a factor of 100,000 for simplification (n = 5–6). *D*, insulin content of isolated control and β-SKO islets (n = 8). All data are represented as mean ± SEM. *p*-values: ∗ < 0.05, ∗∗ < 0.005.

Taken together, manipulation of the diet showed that the hypoglycemic phenotype of β-SKO mice is worsened by increasing the long-term availability of leucine, glutamine, and alanine. Since this effect apparently occurred without increased plasma insulin levels (Fig. 4*C* and S5*C*), we investigated the response of an amino acid challenge in the mouse model. The mice were injected intraperitoneally with the same amino acid mixture as in the diet, and glucose and insulin levels were monitored. Although we observed an acute response of increased blood glucose in all animals, the β-SKO mice exhibited lower values than controls at all time points measured (Fig. 5*A*). Plasma insulin was significantly higher in the β-SKO mice at 10 and 30 min, up to 3 to 4 times higher than in controls (Fig. 5*B*). At 45 min, β-SKO insulin had almost returned to the initial level and did not show a significant difference when compared to controls.

Finally, we investigated differences in secretion and hormone content from isolated islets stimulated with glucose and amino acids. Freshly isolated islets from control and β-SKO mice were cultured overnight, and healthy islets were handpicked and incubated for 1 h in the presence of low (3.3 mM) or high (16.7 mM) glucose alone or in combination with the amino acid mixture of leucine, glutamine and alanine (8). Under low-glucose conditions, insulin levels in the media from β-SKO islets tended to be higher compared to control islets although the difference did not reach statistical significance (Fig. 5*C*). When amino acids were added, insulin secretion from both islet groups increased. However, the stimulated secretory response was clearly more pronounced in β-SKO islets (controls *versus* β-SKO; 6.2 ± 1.2 *versus* 16.0 ± 4.1 (ng/h/ng DNA), *p* = 0.005). Under high-glucose conditions, insulin secretion was similar between groups, except when β-SKO islets with and without amino acid stimulation were compared. In the latter scenario, we observed higher secretion when the amino acid mixture was added to the islets (14.1 ± 5.6 *versus* 6.5 ± 2.8 (ng/h/ng DNA), *p* = 0.02) (Fig. 5*C*). The islet insulin content was similar between the control and β-SKO groups (Fig. 5*D*), implying that differences in secretion could not be explained by different insulin protein expression. In summary, our experiments demonstrated increased sensitivity of β-SKO mice and isolated islets to amino acid-stimulated insulin secretion.

### β-cell SCHAD deficiency alters islet metabolism and is associated with β-cell dedifferentiation

**Figure 6:**
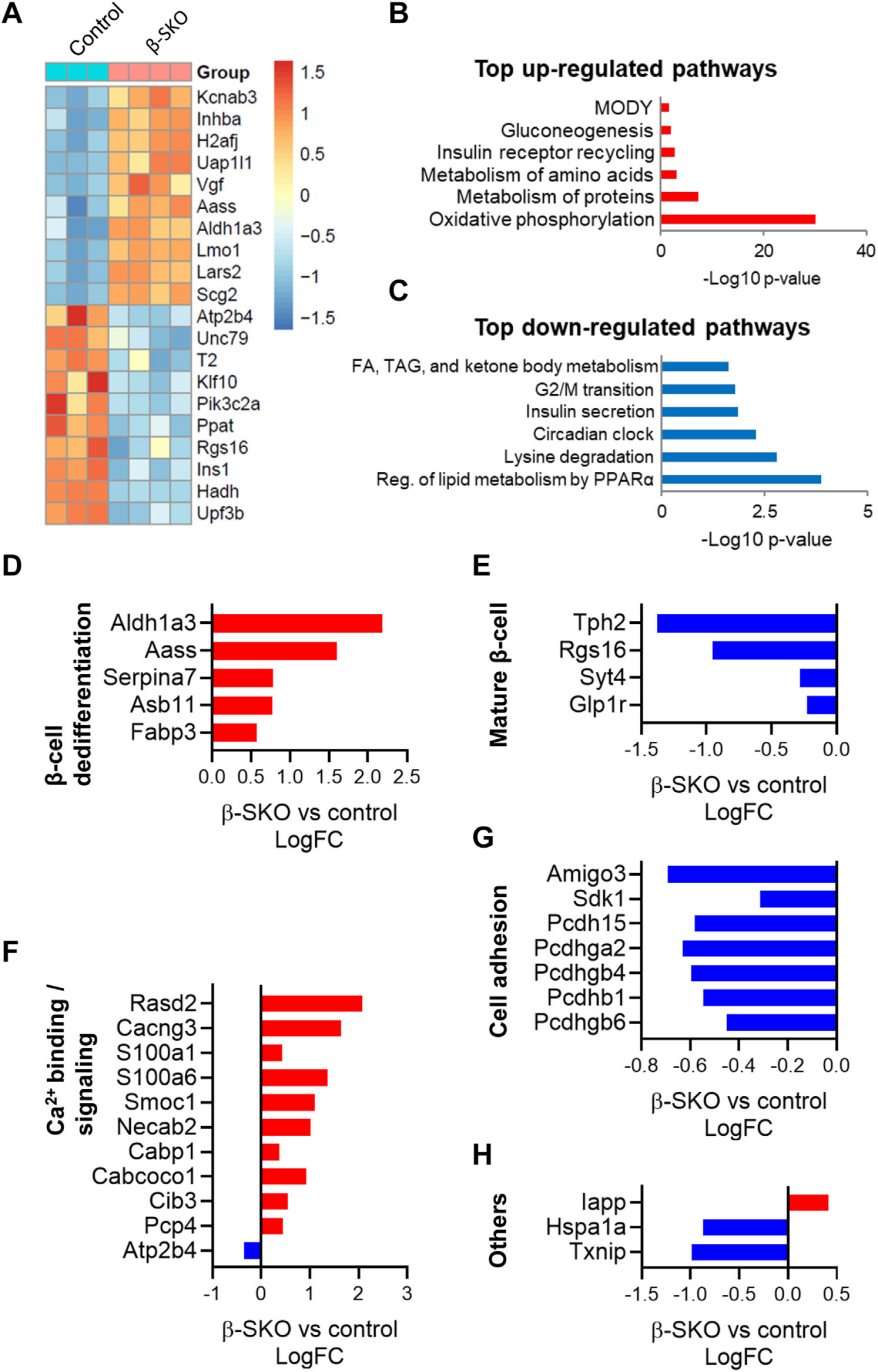
**RNA sequencing of islets isolated from male control and β-SKO mice.***A*, heat map representation of mRNAs differentially expressed between islets isolated from control and β-SKO mice (10 weeks old). Data are shown as Z-scores to indicate the deviation from the group’s mean value. Significantly differentially expressed genes are presented in rows with high to low expression being represented by a change of color from *red* to *blue*. *B*, top upregulated pathways in β-SKO islets identified using over-representation analysis of the top 100 up-regulated genes. Y-axis denotes regulated pathways. X-axis represents their log10 *p*-value. *C*, top downregulated pathways in β-SKO islets identified using over-representation analysis of the top 100 down-regulated genes. Axes as in (*B*). *D*–*H*, differentially expressed genes related to β-cell identity, calcium binding and signaling, and cell adhesion. Gene names are indicated on the *left*. Increased and decreased gene expression are indicated in *red* and *blue* color, respectively. The X-axis represents their log10 *p*-value. FC, fold change.

To determine the effect of β-cell SCHAD deficiency on islet gene expression, we performed RNA sequencing using islet samples from 10-week-old male control or β-SKO mice. Knockout of SCHAD in β-cells was reflected by *Hadh* being among the top down-regulated genes in β-SKO mice (Fig. 6*A*). Additionally, a significant reduction in *Ins1* mRNA expression was consistent with the premise that the *Ins1*-Cre transgene used to target β-cells replaces one *Ins1* allele, thereby inducing a heterozygous gene knockout.

Pathway enrichment analysis of upregulated genes indicated global changes in islet metabolism, such as oxidative phosphorylation, and protein and amino acid metabolism, in β-SKO compared to control islets (Fig. 6*B*). On the other hand, downregulated genes were enriched for pathways associated with the regulation of lipid metabolism, lysine degradation, and circadian clock (Fig. 6*C*). Notably, the insulin secretion pathway was amongst the most down-regulated due to a decrease in the expression of *Pclo, Prkx, Snap25, Glpr1, Prkca* and *Itpr3* genes. Detailed networks of the pathways that are up- and downregulated are shown in Figs. S6 and S7, respectively.

We noted an increase in the expression of a number of genes such as *Aldh1a3* and *Aass* that are associated with β-cell dedifferentiation (Fig. 6*D*) (18, 19). Furthermore, genes involved in β-cell maturation, function, and survival, such as *Tph2* and *Rgs16*, were among the most down-regulated genes in β-SKO islets (Fig. 6*E*) (20, 21). Calcium-related genes made up a large group of differentially expressed genes in β-SKO islets (Fig. 6*F*). This pathway included increased expression of *Rasd2*, a subunit of a voltage-gated Ca^2+^ channel (*Cacng3*), and several calcium-binding proteins (*S100a1, S100a6, Smoc1, Necab2, Cabp1, Cabcoco1, Cib3, Pcp4*). Interestingly, *Atp2b4*, a plasma membrane transporter that removes Ca^2+^ from cells against large concentration gradients, was found to be downregulated. An impact of SCHAD deficiency on islet structure was indicated by the downregulation of the adhesion molecules *Amigo3* and *Sdk1*, several potential calcium-dependent cell-adhesion proteins (*Pcdhga2, Pcdhgb4, Pcdhb1, Pcdhgb6*), and the calcium-dependent cadherin, *Pcdh15* (Fig. 6*G*). We also noted the upregulation of *Iapp* and downregulation of *Txnip* and *Hspa1a*, which may negatively regulate *Iapp* expression and prevent *Iapp* aggregation, respectively (22, 23) (Fig. 6*H*). Overall, the RNA sequencing data suggest that deficiency of SCHAD in β-cells is associated with dedifferentiation and a widespread change in genes relevant to calcium dynamics.

Finally, to directly examine whether Ca^2+^ handling is implicated, we used freshly isolated islets from control or β-SKO mice and stained them with Fluo-Forte, a fluorogenic dye that detects mobilization of intracellular Ca^2+^ (24). Islets were incubated at 3.3 mM glucose with or without the mixture of leucine, glutamine, and alanine used in the previous experiments. There was a robust increase in fluorescent intensity in the β-SKO islets in the presence of amino acids, in contrast to a virtually absent response in control islets (Fig. S8). These data suggest that the difference in amino acid responsiveness between the groups is, at least partly, due to an altered calcium response.

## Discussion

The discovery that the absence of SCHAD enzyme activity can cause CHI (3, 4) was surprising. Pathways of fatty acid degradation had not previously been linked to the disease, and the typical SCHAD-CHI patient does not exhibit the clinical features characteristic for disorders of defective fatty acid oxidation. The question was therefore if the SCHAD protein serves functions outside of fatty acid oxidation and if such functions are specific to the pancreatic β-cell. Notably, SCHAD interacts with many metabolic enzymes, some of which serve tissue-specific functions in the liver (16). However, in terms of glucose homeostasis, the most relevant interaction identified so far is the inhibitory regulation of the ubiquitously expressed enzyme GDH (7, 8). This finding explained, at least partly, the sensitivity to the allosteric activator of GDH, L-leucine, observed in SCHAD-CHI (25).

Nevertheless, SCHAD is a ubiquitously expressed protein, and SCHAD deficiency is also associated with GDH-independent serum and urine changes in metabolites such as 3-hydroxybutyryl-carnitine and 3-hydroxyglutaric acid (4). Moreover, activating mutations in GDH tend to cause hyperammonemia (9), a phenotype generally absent in SCHAD-CHI. Therefore, an unexplored question was whether SCHAD-CHI is caused by a loss-of-function restricted to the β-cell or if other cell types (*e.g.*, hepatocytes) and organ systems are involved. To directly address this question, we created a conditional knockout model of *Hadh* to explore the effects of SCHAD deficiency specifically in β-cells or hepatocytes.

L-SKO mice did not show any phenotype associated with glucose homeostasis, suggesting that the glucose-lowering effect of mutations in the *Hadh* gene is unlikely to be mediated by the liver. Our β-SKO mice, on the other hand, exhibited lower blood glucose than control animals under all tested conditions, including a diminished recovery of glucose during refeeding after fasting. The general hypoglycemia and the stimulated insulin secretion when β-SKO mice or freshly isolated islets were challenged with amino acids indicate that the phenotype of the β-SKO model is similar to that of global knockouts Still, there are some differences. The global SCHADKO mouse displayed improved glucose tolerance whereas our β-SKO animals exhibited either unchanged or slightly impaired tolerance. Moreover, the global SCHADKO mouse had higher basal insulin levels than controls both after 2 and 24 h of fasting (8), while we were not able to detect increased insulin secretion in the β-SKO model, except when the mice were acutely stimulated with amino acids or in isolated β-SKO islets. Methodological differences might explain some of these discrepancies (*e.g.*, oral glucose in (8) *versus* intraperitoneal glucose in the present study; the use of radioimmunoassay in (8) *versus* ELISA in the present study to measure insulin). Thus, in the islet transplantation study reported earlier, we did not observe elevated basal insulin levels in global SCHADKOs or SCHADKO-transplanted mice when compared with controls (14). Indeed, increased insulin levels are also difficult to detect in confirmed CHI patients (26). Nevertheless, as the SCHAD protein is ubiquitously expressed, one possible explanation of the differences in glucose homeostasis between global and β-cell-specific SCHAD knockouts is that other tissues/cell types than the pancreatic β-cell contribute to the phenotype of SCHAD-CHI.

Furthermore, sex-specific differences may also influence the observations in the β-cell-specific KO model. Following refeeding after fasting, plasma insulin and C-peptide levels were reduced in male β-SKO mice compared to littermate controls (Fig. 2, *D* and *E*), reflecting an appropriate physiological response of the endocrine pancreas to the low levels of ambient glucose. In the females, however, the insulin and C-peptide measurements were strikingly similar in the two groups, despite the differences in blood glucose (Fig. S3, *D* and *E*). Interestingly, the elevated insulin/glucose ratio in female β-SKO animals is in line with the phenotype of the global SCHADKO mouse (8). Further studies are warranted to address this intriguing observation.

Feeding a diet enriched in the amino acids leucine, alanine, and glutamine reduced blood glucose levels in β-SKO mice (Fig. 4). Although insulin levels were unaltered in our diet study, there was a considerably stronger increase in plasma insulin in β-SKOs than in controls when the animals were challenged with intraperitoneal injection of amino acids (Fig. 5*B*). Notably, this acute insulin response was accompanied by a steady increase in glucose levels, although always with lower values in the β-SKO animals than in controls (Fig. 5*A*). The concurrent rise in glucose and insulin is not unique to our study as also others have reported that amino acid injection in mice results in increased glucose levels (27, 28). Whether this is potentially due to increased glucagon output from the islets requires further study. Thus, the liver/α-cell axis may act independently of glucose in mice, allowing the amino acid load to counteract the inhibitory effect of glucose on glucagon secretion (28, 29).

The enhanced amino acid-stimulated insulin secretion observed when SCHAD is lacking, can be explained by an inhibitory effect of the SCHAD protein on GDH (8). If SCHAD interferes with the binding of leucine to GDH to inhibit allosteric activation, the absence of SCHAD in β-cells will lead to increased sensitivity to amino acid-induced activation of GDH. It is known that the ability of leucine to activate GDH is inversely correlated to the ATP/ADP ratio, which increases in a high-glucose environment (30, 31). Leucine is therefore limited in its ability to activate GDH in islets stimulated by high glucose while it potently stimulates glutaminolysis under low-glucose conditions. On the other hand, leucine may itself be metabolized and enter the TCA cycle, thereby increasing the ATP/ADP ratio (32). To exclude this possibility, the non-metabolizable leucine analog BCH (33) could be used to test if insulin secretion continues to be augmented under low-glucose conditions.

To gain insights into the changes in gene expression secondary to SCHAD deficiency, we performed RNA sequencing of whole islets. Pathway enrichment analysis identified down-regulation of lipid metabolism-related genes, while those of oxidative phosphorylation and protein metabolism were upregulated in β-SKO animals. This might reflect defects in fatty acid β-oxidation and compensatory mechanisms caused by SCHAD deficiency in affected β-cells. While the majority of the hyperinsulinemic phenotype of SCHAD deficiency may be attributed to its interaction with GDH, a metabolic shift towards amino acid and protein metabolism could be a contributing factor that requires further investigation. We also noted that insulin secretion was among the most downregulated pathways likely due to reduced expression in six genes (*Pclo*, *Prkx*, *Snap25*, *Glpr1*, *Prkca*, *Itpr3*), most of which are associated with calcium-binding or regulation of intracellular calcium (34, 35, 36, 37). These gene expression changes might reflect altered intracellular calcium homeostasis and indicate compensatory mechanisms to modulate insulin secretion from dysregulated β-SKO β-cells. Indeed, the upregulation of several genes involved in calcium-binding and signaling (Fig. 5*F*) in β-SKO islets coupled with direct staining of intracellular Ca^2+^ implicates calcium handling in the phenotype (Fig. S8).

A striking finding in the β-SKO group was that expression of β-cell dedifferentiation markers, such as *Aldh1a3*, were upregulated, while markers of mature β-cells and cell adhesion proteins were amongst the downregulated genes. This gene expression pattern resembled the changes seen in diabetes and in β-cells of the *Abcc8*^−/−^ mouse, a model of K_ATP_ deficiency, and chronic β-cell depolarization (18, 38). However, all gene expression changes were less severe than those of the *Abcc8*^−/−^ mouse. This may be explained by a variety of factors. Firstly, whole β-SKO islets were used in this report compared to purified *Abcc8*^−/−^ β-cells (18). Secondly, the overall phenotype of β-SKO may be milder compared to *Abcc8*^−/−^ mice. Normal calcium levels were observed in the basal state in SCHADKO islets (8) and also in the β-SKO islets (Fig. S8), whereas *Abcc8*^−/−^ islets are constitutively depolarized (18, 39). Therefore, the effect of calcium on calcium-related gene expression may be weaker in SCHAD deficient β-cells. Nevertheless, the overall gene expression pattern of β-SKO islets is consistent with chronic β-cell depolarization and excitotoxicity associated with a hyperinsulinemic phenotype (18). Thus, gene expression changes in the pancreatic islet due to β-cell SCHAD deficiency are consistent with other models of congenital hyperinsulinism and diabetes and may reflect β-cell dysfunction and dedifferentiation due to excitotoxicity.

Finally, we hypothesize that the function of SCHAD in the insulin secretion pathway is to negatively regulate amino acid sensing and amino acid-stimulated insulin secretion. This might ensure appropriate insulin secretion when plasma glucose levels are low and amino acid levels are high (*e.g.*, after a fast or a protein-rich meal). Such a model is consistent with our study in freshly isolated islets where insulin secretion from β-SKO islets was significantly higher than from controls only under low-glucose conditions and upon amino acid stimulation (Fig. 5*C*). In this context it is worth noting that, during embryogenesis, the developing β-cell relies on amino acid sensing to regulate insulin secretion as glucose sensing is not fully established (40). SCHAD expression develops gradually during embryogenesis and peaks in mature islets (41). Perhaps, the postnatal increase in SCHAD expression may be involved in the transition from amino acid to glucose-stimulated insulin secretion. The increase in expression of dedifferentiation markers and reduced expression of maturity genes in β-SKOs suggests SCHAD may be important for regulating cell maturation and identity.

Besides β-cells, α-cells also rely on amino acid sensing to regulate systemic amino acid levels through the liver-α-cell axis (29). The extremely low expression of SCHAD in this islet cell type (42, 43) suggests that α-cells either utilize a SCHAD-independent pathway or have evolved suppressed SCHAD expression to ensure a higher degree of amino acid sensitivity than β-cells. In sum, these data point to a more important role for SCHAD in the β-cell compared to the hepatocyte in the regulation of glucose homeostasis. The versatility of the *Hadh*^lox/lox^ mouse offers a useful model for further dissecting the intra-islet heterogeneity of amino acid sensing as well as exploring the role of SCHAD in amino acid-stimulated secretory pathways beyond the islets.

## Experimental procedures

### Mouse lines and generation of *Hadh*^*lox/lox*^ and tissue-specific SCHAD knockout mice

The whole-body SCHAD knockout mouse (mixed B6/129 background) was originally described by Li *et al.* (8). Heterozygote males and females were bred, and *Hadh*^+/+^ (wildtype) and *Hadh*^−/−^ (SCHADKO) offspring were used for experiments. Genotyping was done as previously reported (8).

For generating conditional SCHADKO mice, LoxP sites flanking exon 3 of the *Hadh* gene were introduced through homologous recombination as indicated in Fig. S1*A*. *Hadh*^tm1a(EUCOMM)Hmgu^ targeted mouse embryonic stem cell clones of the C57BL/6N cell line JM8A1.N3 were produced by EuCOMM (European Conditional Mouse Mutagenesis Program, Helmholtz Zentrum München, Neuherberg, Germany), using a L1L2_Bact_P targeting vector. The vector was composed of an FRT-flanked selection cassette upstream of the LoxP-flanked exon 3 of the mouse *Hadh* gene. The selection cassette contained a LacZ sequence downstream of the first FRT site, followed by a LoxP site, neomycin under the control of a human beta-actin promoter, and an SV40 polyA signal.

The targeted embryonic stem cells were injected into mouse blastocysts for the generation of chimeric mice at Beth Israel Deaconess Transgenic Core Facility (Boston, MA). Chimeric mice were identified by coat color and subsequently crossed with C57BL/6J mice to produce transgenic offspring. For removal of the FRT-flanked selection cassette, the offspring were crossed with ROSA26::FLPe knock-in animals (Jackson Laboratory, #009086). Successful generation of *Hadh*^*lox/lox*^ mice was confirmed by sequencing of the *Hadh* exon 3 region. The transgenic mice were then crossed with either B6(Cg)-*Ins1*^*tm1.1(cre)Thor*^/J (Ins1-Cre) mice (Jackson Laboratory, #02680) or Alb-Cre mice (Jackson Laboratory, #003574) to generate beta-cell (β-SKO) or hepatocyte (L-SKO)-specific SCHAD knockout animals.

### Genotyping of *Hadh*^*lox/lox*^ mice and confirmation of cre-mediated excision of *Hadh* exon 3

Genotyping of *Hadh*^*lox/lox*^ mice was done by PCR using the reverse primer P1 (5′-ATTGGAGGTGTGGCCTTAGAGGAA-3′), positioned in intron 3 downstream of the second LoxP site, and forward primer P2 (5′-GGAGATGAGTTTGTGGAGAAGACC-3′) in exon 3 (Fig. S1*A*). The amplification results in products of 570 bp for the wildtype allele and 600 bp for the floxed allele.

For validation of Cre-mediated recombination in β-KO islets and L-KO liver, the forward primer P3 (5′-GGTGTGTTTCTCTGTGGTAAGGAC-3′), positioned upstream of the FRT site and first LoxP site, was included in the reaction (Fig. S1*A*). Recombination removes *Hadh* exon 3, which contains the binding site for P2. Thus, a duplex PCR using P1, P2, and P3 results in a 600 bp P1-P2 product before recombination and a 450 bp P1-P3 product when exon 3 has been excised.

### Animal care and diet

Animal studies were approved by the Institutional Review Board of Joslin Diabetes Center and were in accordance with National Institutes of Health (NIH) guidelines. Mice were housed on a 12-h light/dark cycle (22 °C room temperature), with a*d libitum* access to water and normal chow (Rodent 5058; LabDiet), OpenStandard (CD1; Research Diets, #D11112201), custom control (CD2; Research Diets, #D18071302) or custom amino acid-enriched diet (ED; Research Diets, #D18071301). Details about diets CD1, CD2 and ED are given in Table S1. As there were no significant phenotypic differences between the two control groups, data from CD1-fed and CD2-fed mice were combined as one pooled control group under the term normal diet (ND).

### *In vivo* metabolic testing

Plasma glucose and hormone levels were obtained from fed, overnight (16 h) fasted, and refed mice. Samples for refed conditions were collected 4 h after re-feeding the regular chow diet. Mice were housed in individual cages for refeeding, and the weight of the food pellets was measured at t = 0 and 4 h to measure food consumption.

Mice were subjected to glucose tolerance tests (GTT), insulin tolerance tests (ITT), and glucose-stimulated insulin secretion assays (GSIS) as described previously (44). The animals were fasted for 16 h before GTT and GSIS, or 3 h for ITT. Two g glucose, 3 g glucose, or 1 U insulin per kg body weight were administered by intraperitoneal injection for GTT, GSIS, and ITT, respectively. Whole-blood glucose levels were obtained during GTT and ITT at t = 0, 15, 30, 60, and 120 min. For GSIS, whole blood was collected at t = 0, 2, and 5 min.

Glucose was measured from blood collected from the tail vein using a standard glucometer. Whole-blood samples were centrifuged, and serum/plasma was stored at −20 °C until assayed for insulin (Crystal Chem, #90080) and C-peptide (Crystal Chem, #90050) levels by ELISA. For glucagon ELISA (Crystal Chem, #81518), whole blood was collected in chilled tubes containing heparin, centrifuged, and plasma was immediately used for glucagon measurement.

For assessment of acute insulin secretion in response to an amino acid challenge, female mice (aged 10–12 weeks) were fasted for 16 h. A mixture of leucine (0.46 mM; Millipore Sigma, #8912), glutamine (2 mM; Millipore Sigma, #G8540), and alanine (1.25 mM; Millipore Sigma, #A7469), described previously (8), was diluted in PBS buffer and administered intraperitoneally (1.5 g total amino acid/kg body weight). Blood samples were obtained from the tail prior to (0 min) and 10, 30, and 45 min after injection for measurement of insulin levels using an ELISA as described above.

### Islet isolation and functional analyses

Islets were isolated, pooled, and cultured overnight from 10-week-old male and female mice as described in (45). For the insulin secretion assay, 20 handpicked and size-matched islets were cultured in Krebs-Ringer bicarbonate buffer for 1 h before stimulation with either low (3.3 mM) or high (16.7 mM) glucose alone or in combination with the above-mentioned amino acid mixture.

The stimulation buffer was then collected for measurement of secreted insulin. The islet pellet was sonicated for acid ethanol extraction and subsequent measurement of intracellular hormone content by ELISA. Finally, DNA was extracted from the islet lysates and spectrophotometric measurements of the DNA content used for data normalization.

For assessment of Ca^2+^ mobilization, freshly isolated islets were handpicked and placed in groups of 20 into a black-wall 96-well plate. They were stained for 45 min with Fluo-Forte calcium-binding dye (ENZO, #51016) diluted in Krebs Ringer Buffer solution (137 mM NaCl, 4.8 mM KCl, 1.2 mM KH_2_PO_4_, 1.2 mM MgSO_4_, 2.5 mM CaCl_2_, 5 mM NaHCO_3_, 16 mM HEPES, 0.1% BSA), followed by washing for 15 min with the same solution. The islets were then stimulated with 3.3 mM glucose alone or in combination with the amino acid mixture described earlier. Images were taken 10 s after the addition of the solution using an AxioZoom V16 microscope (Zeiss) with excitation and emission wavelengths of 495 nm and 520 nm, respectively.

### Western blot

Tissue samples were homogenized in RIPA buffer containing proteinase and phosphatase inhibitors. Protein concentrations were determined by Pierce BCA assay (ThermoFisher Scientific, #23225) followed by standard western blotting. Proteins were detected using the following primary antibodies: SCHAD (Genetex, GTX105167, rabbit polyclonal), α-tubulin (Abcam, ab6046, rabbit polyclonal), and β-actin (Santa Cruz Biotechnology, sc-47778 HRP, mouse monoclonal). Blots were developed using ECL (ThermoFisher Scientific, #32106).

### Histology

Pancreas samples were harvested from *Hadh*^*lox/lox*^, SCHADKO, and β-SKO mice and immediately fixed in 4% paraformaldehyde solution overnight at 4 °C. Samples were then stored in PBS at 4 °C until processing and paraffin embedding. Immunostaining (5-μm tissue sections) for SCHAD and insulin was performed as previously described (14). For glucagon and SST staining, the sections were incubated overnight at 4 °C with mouse monoclonal anti-glucagon antibody (Sigma-Aldrich, G2654; 1:8000) or rat monoclonal anti-SST antibody (Abcam, ab30788; 1:500), followed by incubation for 1 h at room temperature with secondary antibody 1:400 from Jackson ImmunoResearch Laboratories (glucagon: #715-586-151; SST: #712-586-153).

### RNA sequencing and analysis

Islets were isolated from male, 10-week-old control, and β-SKO mice (45). To increase the amount of extractable RNA, islets from two animals of the same genotype were pooled. Three control (n = 6 animals) and four β-SKO (n = 8) pooled samples were generated. Islet pellets were lysed in RLT buffer containing 10 μl/ml β-mercaptoethanol (RNeasy Plus Mini Kit, Qiagen) and submitted to HudsonAlpha Institute for Biotechnology for RNA isolation and sequencing (paired-end, 50 bp). Data analysis was carried out by the Bioinformatics and Biostatistics Core at Joslin Diabetes Center as described in (46). To reduce the number of false positives, we filtered genes showing an FDR<0.25 and performed pathway analyses using ConsensusPathDB (47) with default settings.

### Statistics

Data are shown as mean ± SEM. All statistical analyses were performed using GraphPad Prism 9 (www.graphpad.com). For differences between control and β-SKO mice, or between two conditions of the same genotype, the nonparametric Mann-Whitney *U* test was performed. Statistical interactions between more than two groups were analyzed by two-way ANOVA. A *p* value <0.05 was considered statistically significant.

## Data availability

All data are contained within the article and supporting information.

## Supporting information

This article contains supporting information.

## Conflict of interest

The authors declare that they have no conflicts of interest with the contents of this article.
