# Supplementary material for: Deficiency of the metabolic enzyme SCHAD in pancreatic β-cells promotes amino acid–sensitive hypoglycemia
Source: J Biol Chem. 2023 Jun 29;299(8):104986. doi: 10.1016/j.jbc.2023.104986 (PMC10407745; doi:10.1016/j.jbc.2023.104986)
Supplement: Supporting Figures S1–S8 and Table S1 [file mmc1.pdf]

Suppl. Fig. S1

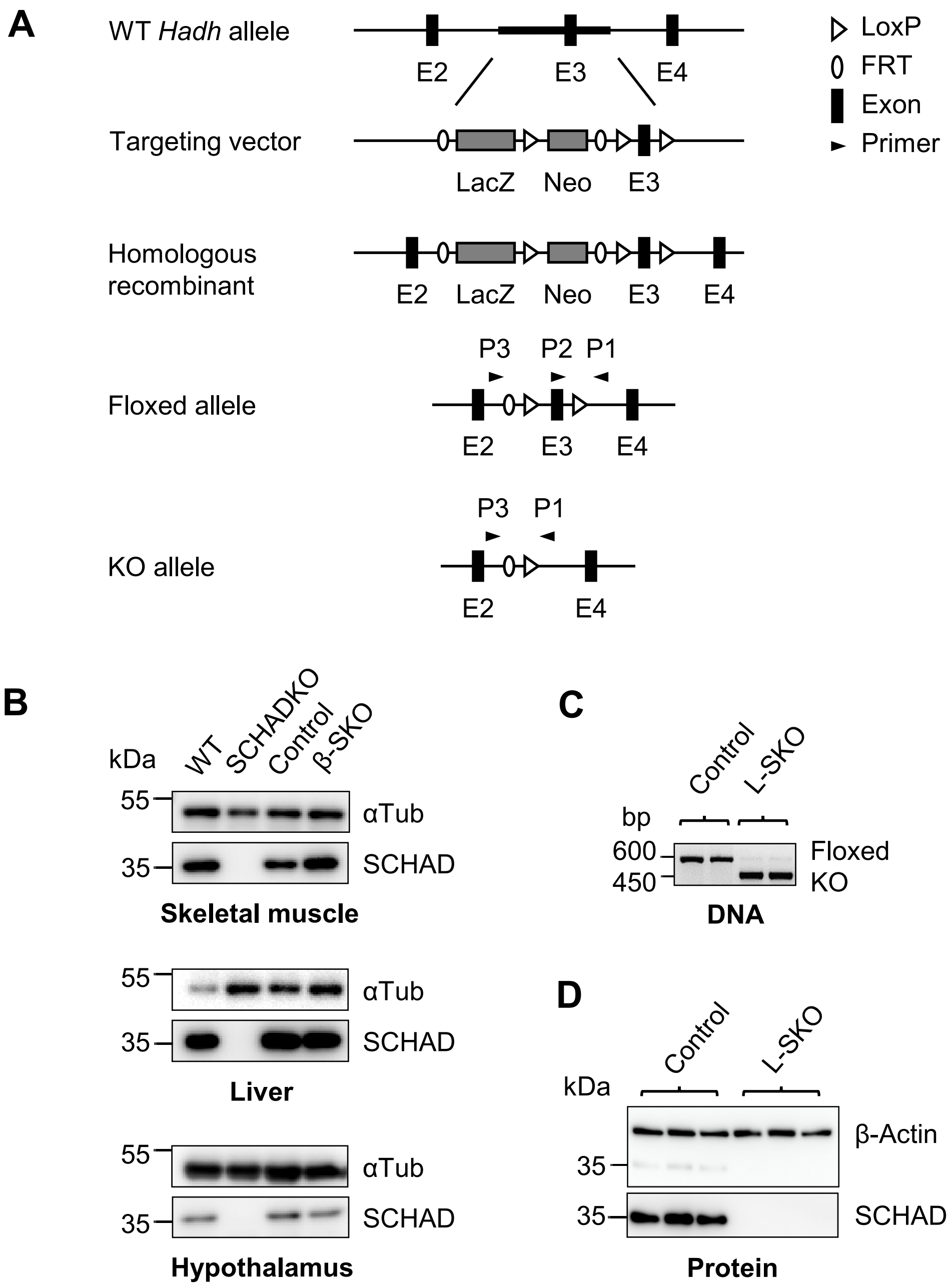

Supplementary Figure S1 – Targeting strategy for disrupting SCHAD expression and validation of β-SKO and L-SKO mice. **(A)** The targeting vector replaced the endogenous exon 3 (E3) with a LoxP-flanked exon 3 downstream of a LacZ and neomycin (Neo) cassette flanked by FRT sites. Flipase (Flp)-dependent excision of the LacZ and Neo cassette yielded the floxed allele, followed by Cre-mediated recombination to generate the knockout allele. The location of binding sites for PCR primers is schematically indicated for the floxed and knockout allele. **(B)** Western blots for detection of SCHAD protein expression in lysates of muscle, liver and hypothalamus of littermate control and β-SKO mice. Lysates from wildtype mice (WT) and the general *Hadh* knockout mouse (SCHADKO) were included as positive and negative controls, respectively. Detection of tubulin alpha-1A chain (αTub) served as loading control. **(C)** PCR assay for detection of the floxed and knockout *Hadh* alleles in liver DNA samples of control and L-SKO mice. Recombination was assessed using the primers P1, P2 and P3 indicated in (A). **(D)** Western blot for detection of SCHAD protein expression in liver samples of littermate control and L-SKO mice. Detection of β-actin served as loading control.

Suppl. Fig. S2

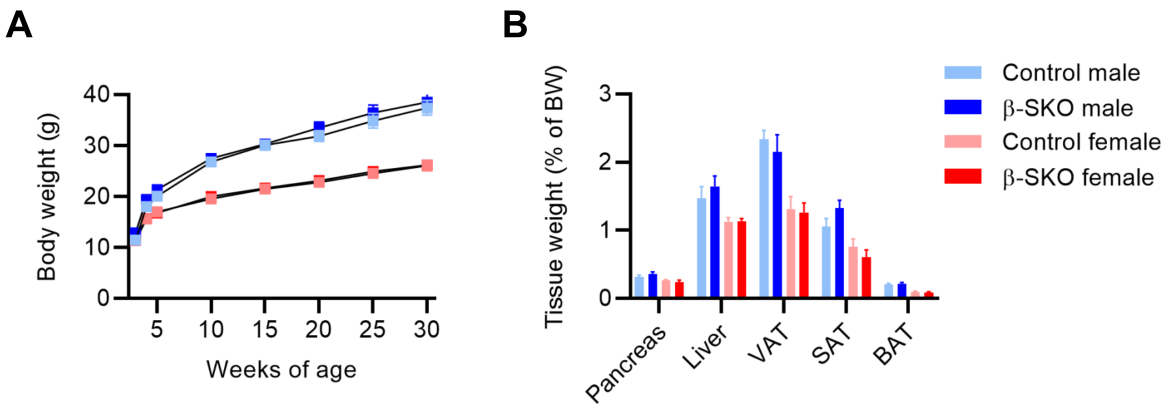

Supplementary Figure S2 – Bodyweight development and organ weights of β-SKO mice.

(A) Bodyweight development of male and female control and β-SKO mice (n=10-12). **(B)** Tissue weights of pancreas, liver, visceral (VAT), subcutaneous (SAT) and brown (BAT) adipose tissue as percentage of bodyweight of 40-week-old male and female control and β-SKO mice (n=10-12). All data are represented as mean ± SEM.

Suppl. Fig. S3

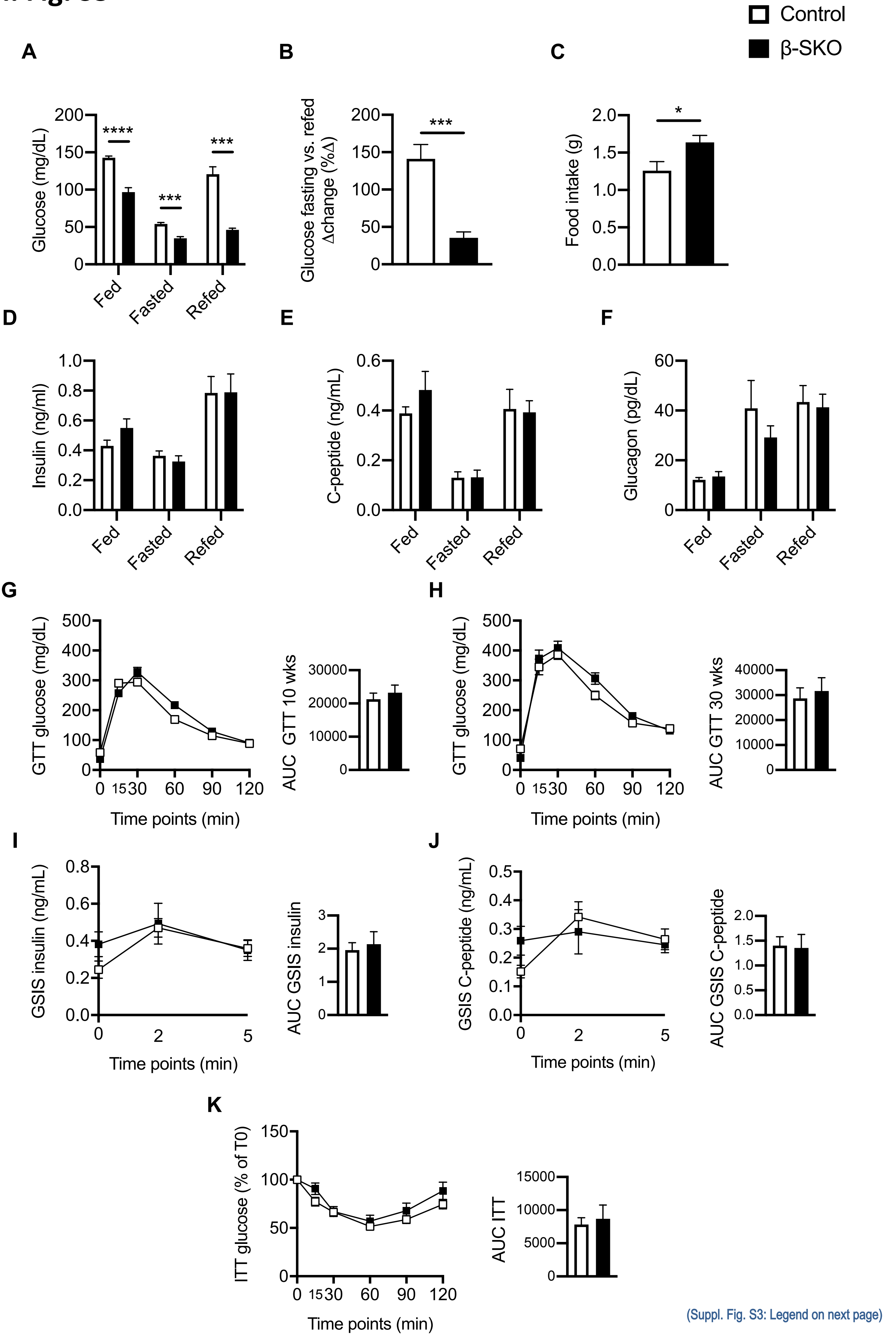

(Suppl. Fig. S3: Legend on next page)

Supplementary Figure S3 – Glucose homeostasis and insulin tolerance in female control and β-SKO mice.

(A) Plasma glucose in fed, 16-hour fasted and refed 13-week-old mice (n=7-10). Data for control vs. β-SKO animals: random-fed, 142.7 ± 2.4 vs. 96.7 ± 5.9 mg/dL; 16 h fasted: 54.1 ± 1.9 vs. 34.9 ± 2.3 mg/dL; 4 h refed: 120.6 ± 10 vs. 46.3 ± 2.1 mg/dL. **(B)** Difference (Δ) in percentage change of fasted vs. refed plasma glucose (n=8). Data for control vs. β-SKO animals: %Δ 141 ± 19.3 vs. 30 ± 9.4 mg/dL. **(C)** Food intake during the 4-hour refeeding period (n=7-8). The intake was mildly increased in the β-SKO females. Data for control vs. β-SKO animals: 1.3 ± 0.1 vs. 1.6 ± 0.09 g. **(D-F)** Plasma insulin (n=8-10), C-peptide (n=8-10) and glucagon (n=7-8) in fed, 16-hour fasted and refed control and β-SKO mice (13 weeks old). **(G)** GTT and area under the curve AUC of 10-week-old control and β-SKO mice (n=12). **(H)** Same as (G) for 30-week-old mice (n=11-12). **(I)** GSIS and AUC of 12-week-old control and β-SKO mice (n=10). **(J)** Plasma C-peptide levels for the mice in (I) (n=10). **(K)** ITT and AUC of 10-week-old control and β-SKO mice (n=11-12). All data are represented as mean ± SEM.

Suppl. Fig. S4

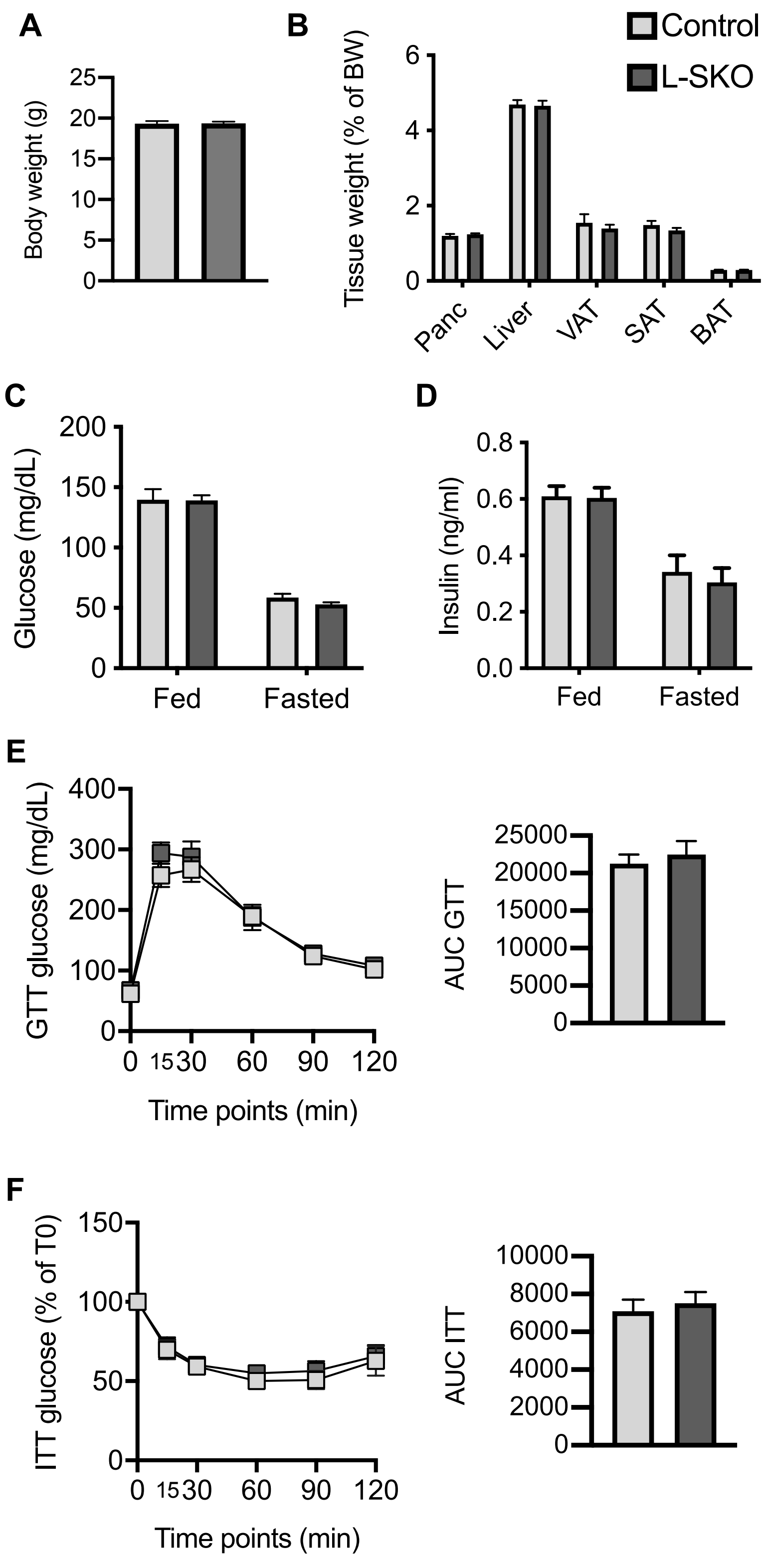

Supplementary Figure S4 – Bodyweight and glucose homeostasis of female control and L-SKO mice.

(A) Bodyweight of 10-week-old control and L-SKO mice (n=9-10). **(B)** Tissue weights of pancreas, liver, visceral (VAT), subcutaneous (SAT) and brown (BAT) adipose tissue as percentage of bodyweight of 14-week-old control and L-SKO mice (n=9-10). **(C-D)** Plasma glucose (n=6-10) and insulin (n=5-10) in 12-week-old control and L-SKO mice. **(E)** GTT and AUC of 10-week-old control and L-SKO mice (n=10). **(F)** ITT and AUC of 10-week-old control and L-SKO mice (n=9-10). All data are represented as mean ± SEM.

Suppl. Fig. S5

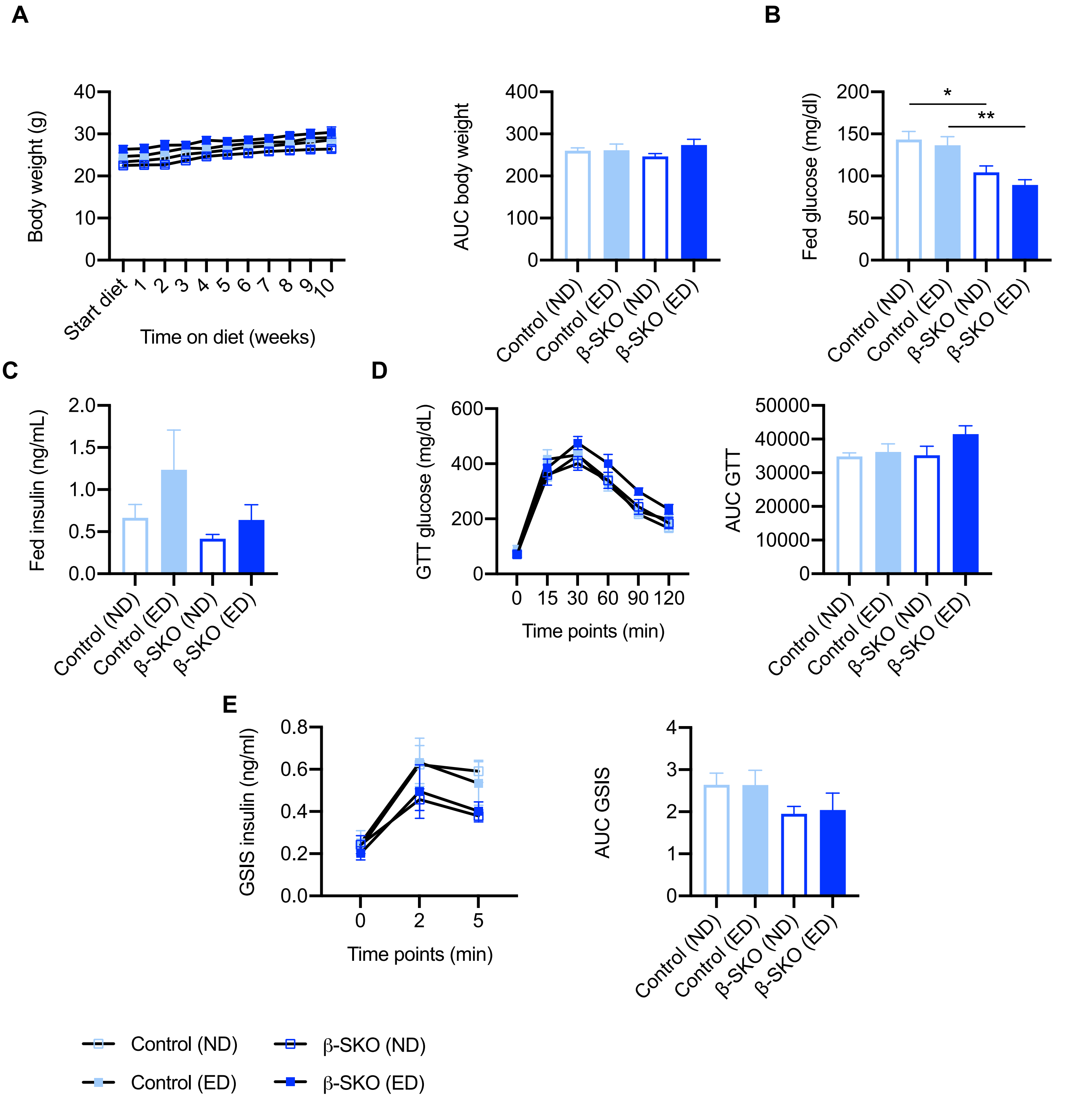

Supplementary Figure S5 – Bodyweight development, random fed plasma glucose, GTT and GSIS of male control and β-SKO mice fed an amino acid-enriched diet.

(A) Bodyweight development and AUC of male control and β-SKO mice over the course of 10 weeks on amino acid-enriched diet (ED) compared with normal diet (ND) (n=6-9). **(B)** Random fed plasma glucose levels of male control and β-SKO mice fed ED or ND for 12 weeks (n=6-9). **(C)** Plasma insulin levels for the mice in (B) (n=6-9).**(D)** GTT and AUC of male control and β-SKO mice fed ED or ND for 17 weeks (n=6-9). **(E)** GSIS and AUC of male control and β-SKO mice fed ED or ND for 14 weeks (n=4-9). All data are represented as mean ± SEM.

Suppl. Fig. S6

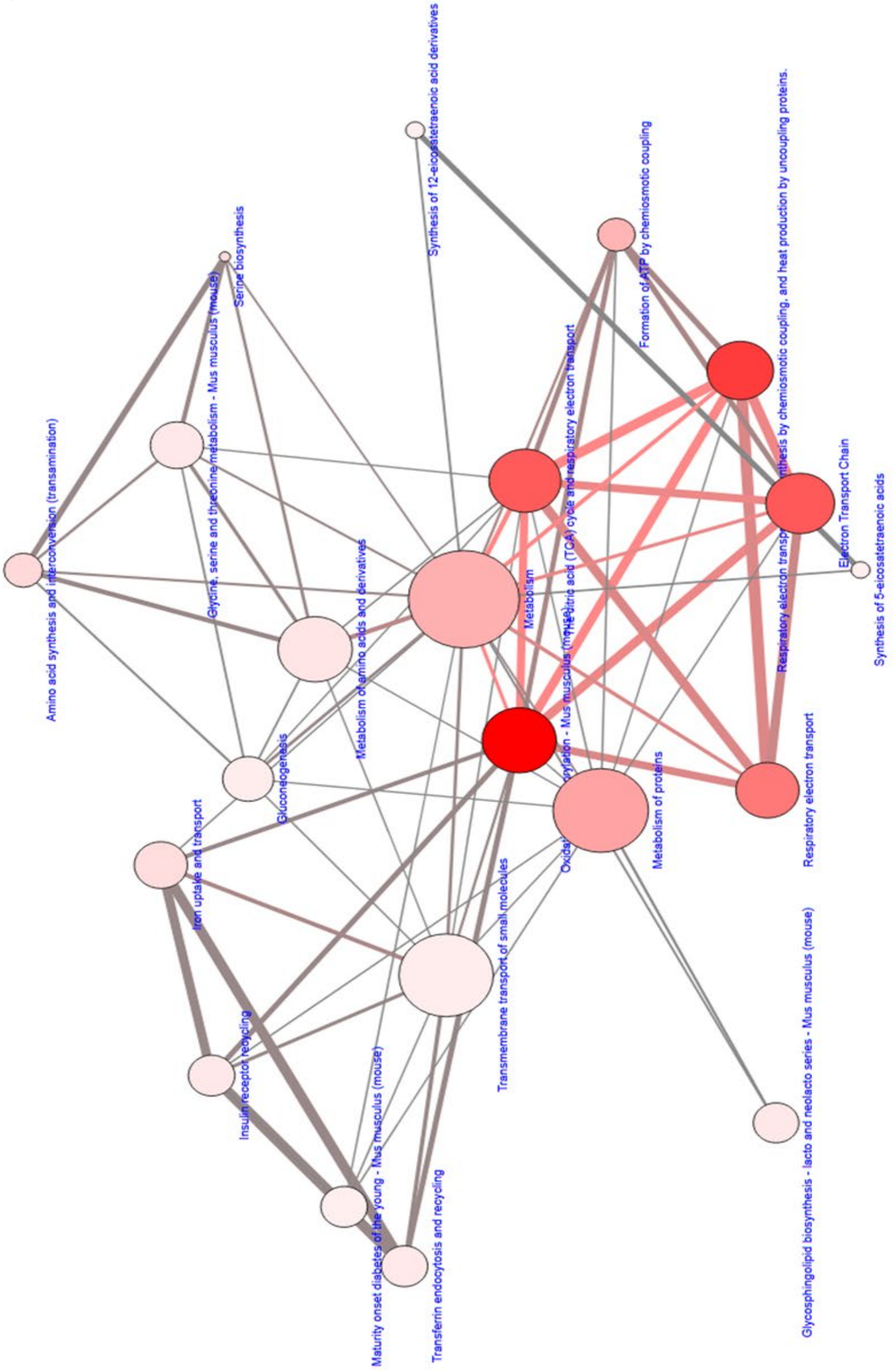

Supplementary Figure S6 – Network of over-represented pathways corresponding to up-regulated genes in β-SKO islets.

The network was based on data from RNA sequencing. To reduce the number of false positives, only results with and false discovery rate (FDR) less than 0.25 are shown.

Suppl. Fig. S7

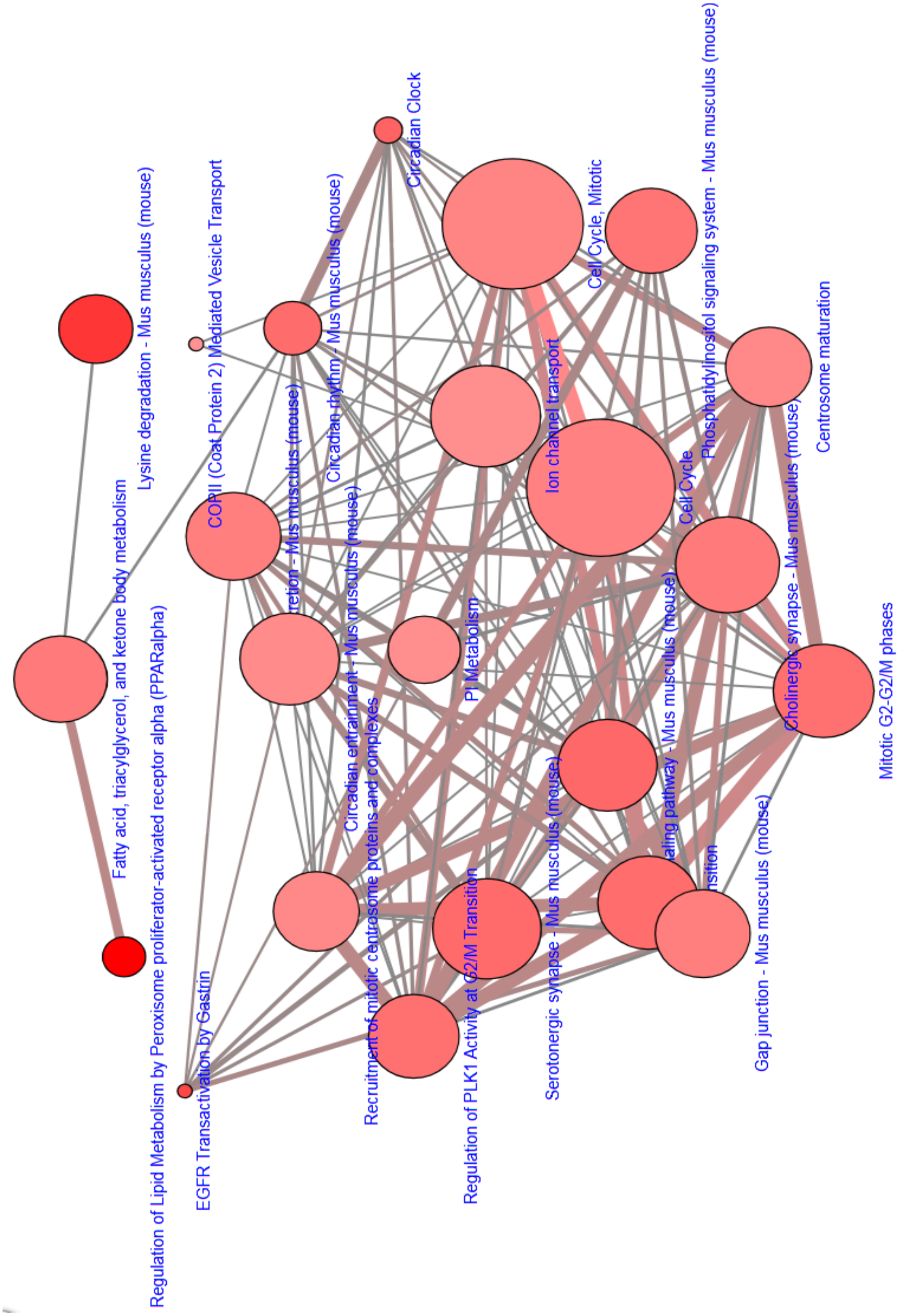

Supplementary Figure S7 – Network of over-represented pathways corresponding to down-regulated genes in β-SKO islets.

The network was based on data from RNA sequencing. To reduce the number of false positives, only results with and false discovery rate (FDR) less than 0.25 are shown.

Suppl. Fig. S8

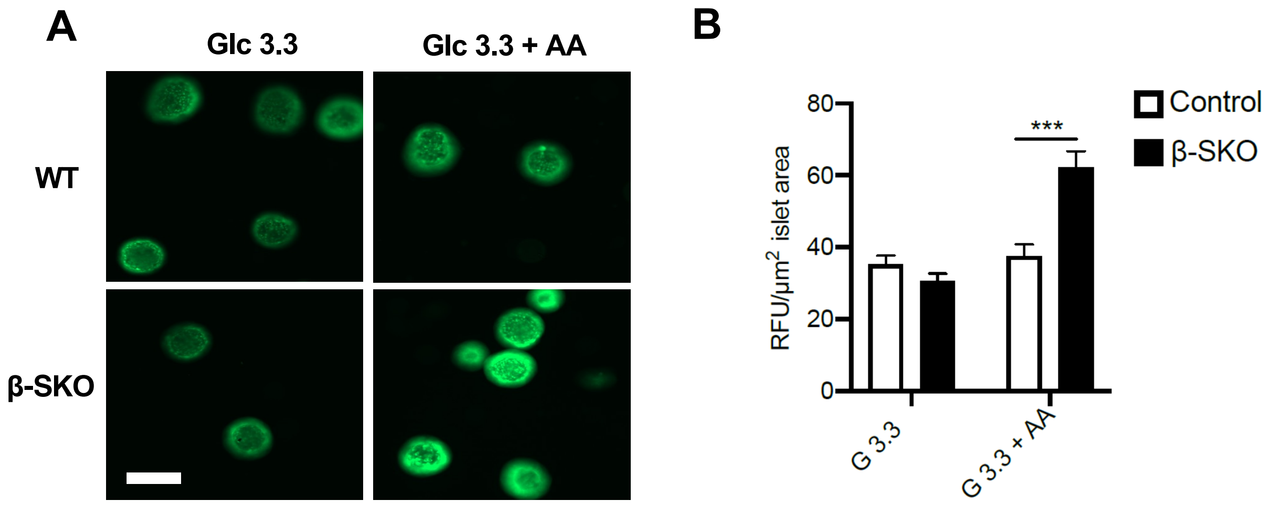

Supplementary Figure S8 – Effect of amino acids on intracellular calcium mobilization.
(A) Representative fluorescent images (520 nm emission) of intact islets from β-SKO and control mice incubated with Fluo-Forte Ca+2 binding dye in 3.3 mM glucose (G3.3) with and without amino acid mixture (AA). Scale bar is 100 μm for all images. **(B)** Quantification of fluorescent intensity of the images in A. *** < 0.001.

Suppl. Table 1

Designation of diet	OpenStandard		Custom control		Amino acid-enriched	
Abbreviation	CD1		CD2		ED	
Research Diets Product #	D11112201		D18071301		D18071302	
**Composition**	***g%***	***kcal%***	***g%***	***kcal%***	***g%***	***kcal%***
**Protein**	**19**	**20**	**18**	**20**	**24**	**26**
**Carbohydrate**	**63**	**65**	**67**	**71**	**61**	**65**
**Fat**	**7**	**15**	**4**	**9**	**4**	**9**
**Total**		**100**		**100**		**100**
**Kcal / g**	**3.81**		**3.69**		**3.69**	
**Ingredient**	***g***	***kcal***	***g***	***kcal***	***g***	***kcal***
**Casein**	200	800	200	800	200	800
**L-Cystine**	3	12	3	12	3	12
**Leucine**	0	0	0	0	16.6	66.4
**Glutamine**	0	0	0	0	39.8	159.2
**Alanine**	0	0	0	0	5.3	21.2
**Corn Starch**	381	1524	442.7	1770.8	381	1524
**Maltodextrin 10**	110	440	110	440	110	440
**Dextrose**	150	600	150	600	150	600
**Cellulose, BW200**	75	0	75	0	75	0
**Inulin**	25	37.5	25	37.5	25	37.5
**Soybean Oil**	70	630	42.6	383.4	42.6	383.4
**Mineral Mix S 10026 (without Ca, P, or K)**	10	0	10	0	10	0
**Dicalcium Phosphate**	13	0	13	0	13	0
**Calcium Carbonate, Light, USP**	5.5	0	5.5	0	55	0
**Potassium Citrate, 1 H2O**	16.5	0	16.5	0	16.5	0
**Vitamin Mix V10001**	10	40	10	40	10	40
**Choline Bitartrate**	2	0	2	0	2	0
**Yellow Dye #5, FD&C**	0.025	0	0	0	0.05	0
**Red Dye #40, FD&C**	0	0	0.05	0	0	0
**Blue Dye #1, FD&C**	0.025	0	0	0	0	0
**Total**	1071	4084	1105	4084	1105	4084
**Leu (g/4084 kcal)**	16.5		16.5		33.1	
**Glu/gln (g/4084 kcal)**	39.8		39.8		79.6	
**Ala (g/4084 kcal)**	5.3		5.3		10.6	

Supplementary Table 1 – Composition of control diets and the custom-made amino acid-enriched diet.

ED contained twice the normal content of the amino acids alanine, glutamine, and leucine which adds a total of 246.8 kcal per kg to the diet. To ensure that the overall caloric content of ED equaled the caloric content of CD1, the fat content was adjusted from 15 to 9 kcal%. Since the amount of fat was reduced in ED, a second custom control diet (CD2) with 9 kcal% from fat was included in the study. To ensure that the overall caloric content of CD2 equaled that of CD1, the kcal% from carbohydrates was increased from 65 to 71 kcal%.
